# The Flux of *Euglena gracilis* Cells Depends on the Gradient of Light Intensity

**DOI:** 10.1371/journal.pone.0168114

**Published:** 2016-12-29

**Authors:** Takuma Ogawa, Erika Shoji, Nobuhiko J. Suematsu, Hiraku Nishimori, Shunsuke Izumi, Akinori Awazu, Makoto Iima

**Affiliations:** 1 Graduate School of Science, Hiroshima University, Higashi-Hiroshima, Hiroshima, Japan; 2 Meiji Institute for Advanced Study of Mathematical Sciences, Meiji University, Tokyo, Japan; 3 Graduate School of Advanced Mathematical Sciences, Meiji University, Tokyo, Japan; Bascom Palmer Eye Institute, UNITED STATES

## Abstract

We have quantified the photomovement behavior of a suspension of *Euglena gracilis* representing a behavioral response to a light gradient. Despite recent measurements of phototaxis and photophobicity, the details of macroscopic behavior of cell photomovements under conditions of light intensity gradients, which are critical to understand recent experiments on spatially localized bioconvection patterns, have not been fully understood. In this paper, the flux of cell number density under a light intensity gradient was measured by the following two experiments. In the first experiment, a capillary containing the cell suspension was illuminated with different light intensities in two regions. In the steady state, the differences of the cell numbers in the two regions normalized by the total number were proportional to the light difference, where the light intensity difference ranged from 0.5–2.0 *μ*mol m^−2^ s^−1^. The proportional coefficient was positive (i.e., the bright region contained many microorganisms) when the mean light intensity was weak (1.25 *μ*mol m^−2^ s^−1^), whereas it was negative when the mean intensity was strong (13.75 *μ*mol m^−2^ s^−1^). In the second experiment, a shallow rectangular container of the suspension was illuminated with stepwise light intensities. The cell number density distribution exhibited a single peak at the position where the light intensity was about *I*_*c*_ ≃ 3.8 *μ*mol m^−2^ s^−1^. These results suggest that the suspension of *E. gracilis* responded to the light gradient and that the favorable light intensity was *I*_*c*_.

## 1 Introduction

*Euglena gracilis* is a unicellular flagellated photosynthetic alga; the body is approximately 10 *μ*m wide and 50–100 *μ*m long. *E.gracilis* carries a stigma (eyespot) in the anterior part, which is red owing to carotenoid pigments contained in the body termed pigment granules. Individuals of *E. gracilis* respond to light via the shading of the stigma onto the paraflagellar body (PFB) at the base of the flagellum. The photo-response behavior of *E. gracilis* can be categorized into phototaxis, photokinesis, and photophobic response [1].

Through phototaxis, *E.gracilis* swims toward a light source if the intensity is less than a critical value (positive phototaxis) and swims away if the intensity is above the critical value (negative phototaxis) [2]. In this paper, we define the phototaxis of the cell flux as photoresponse motion in which the mean swimming velocity is parallel to the light vector according to Vincent and Hill [3]. Häder et al. [4] measured the phototaxis of *E. gralicis* for several illuminances of white light and found that the phototaxis behavior is almost marginal around 250 lx, which is equivalent to 1.05 Wm^−2^ according to the conversion relationship. Giometto et al. [5] measured the phototaxis exhibited under the light distribution generated by a point light source. *E. gracilis* also exhibits a taxis for polarized light [6, 7].

Photokinesis refers to the phenomenon that the linear swimming velocity depends on the light intensity. Noda investigated the swimming velocity of *E. gracilis* under light intensities ranging from 200–2500 lx (S. Noda, Master thesis in Hiroshima University, 2012). It was found that the swimming speed was approximately 60 *μ*m s^−1^ if the light intensity was less than 1000 lx but that it increased as the light intensity increased, with a maximum speed of approximately 90 *μ*m s^−1^ at 2500 lx.

Photophobic response is a light-induced shock response that occurs when the illumination intensity around the microorganism is suddenly changed. Matsunaga et al. [8] measured the statistics of the swimming direction change under sudden temporal change of the light intensity. They measured both the “step-up” change; i.e., the increase of light intensity from the no light condition, and the “step-down” intensity; i.e., the decrease the light intensity to the no light condition. They also defined a photophobic index and measured the index for light with different wavelengths (260nm-520nm) and intensities (0.3–100 *μ*mol m^−2^ s^−1^) and found that the index increased for light intensities in the range 0.1–10 *μ*mol m^−2^ s^−1^, although the detailed behavior depended on the wavelength. In addition, Iseki et al. [9] identified the flavoprotein responsible for the “step-up” responses.

Photomovements of *E. gracilis* cause an interesting macroscopic pattern: bioconvection [3, 10–14]. If a suspension of *E. gracilis* is illuminated from the bottom with strong light, the pattern is spatially localized [15, 16]. Such localized structures can be classified into two groups: one is a single region of high cell density sandwiched with two counter-rotating convection rolls (bioconvection unit), and the other is a traveling wave in a confined region (localized traveling wave). These elementary structures can be obtained in an annular container [15].

Strong illumination from below is a necessary condition for generating these localized bioconvection patterns. Conversely, if the light intensity is weak, the localization pattern is not observed [17]. Whereas a photo response behavior is likely responsible for the localization mechanism, the above-mentioned photomovements appear to be insufficient to describe the observed localization patterns. In particular, movement of the microorganisms owing to phototaxis is parallel to the light vector. In a vertical light vector setup, this is important for the microorganisms to accumulate near the surface and to cause Rayleigh-Taylor instability [18]; however, macroscopic horizontal flux of the cell density to cause the observed spatial localization is not included in this effect.

Vincent and Hill [3] analyzed a hydrodynamic bioconvection model generated by positive phototaxis, where the suspension was illuminated from above. In their model, the phototaxis term induced the cell density flux in the vertical direction alone. The scattering of light has been considered in bioconvection illuminated from above by Ghorai and his coworkers [19, 20]; however, the averaged cell density flux owing to the phototaxis was directed vertically as in the Vincent and Hill model. It therefore appears that localized convection patterns require another photomovement that might be lateral to enhance spatial disturbance.

In this study we assume that the lateral movement is due to photomovement determined by the light gradient, by which an effective accumulation of individuals is possible because when the suspension is illuminated from below, the region near the surface that is *Euglena*-rich is darker compared with other regions. Comparatively, Williams and Bees [21] included an effct of the photomovement owing to the light gradient on the torque applied in the microorganism cell, assuming that the individual cell was able to detect the light gradient. In addition, studies of conditions such as the spatial light trap [7] or photophobic response [8] have suggested some responses of *E. gracilis* to a light gradient.

Giometto et al. [5] obtained an equation of the cell density that includes a spatial potential to cause a population velocity in the direction of the light gradient. However, their measurements are performed under the illumination of point light source. So the light vector is not vertical except the onset of the light source, thus, their equation can not be applied directly to the configuration of the spatially homogeneous illumination (flat light source) which is related to many problems including bioconvection problems. An equation to describe the flux of cells under the light gradient generated by flat light sources will be useful, however, there no direct measurements of the cell density flux of *E. gracilis* under a light gradient have been reported. Here, we focus on the macroscopic behavior of the flux of cell number density (referred to as ‘cell density’ hereafter) and present evidence that the light gradient actually causes a bias of the cell density within heterogeneous illumination. In particular, in this study we measured the photomovement of *E. gracilis* owing to a light gradient. After summarizing the experimental setups below (section 2), we show that the flux of the cell density comprises the sum of diffusion and of another flux that is proportional to the light gradient and the mean cell density (section 3). We estimate the coefficients of the flux as a function of the mean light intensity (section 4) and discuss the observed photomovement behavior in the context of other phenomena such as chemotaxis (section 5).

## 2 Experimental Setups

*E. gracilis* was pre-cultured using Koren-Hutner medium for 2–4 weeks with continuous light illumination at room temperature. Then, the cells were inoculated into 1 g/L HYPONeX solution with periodic light illumination (14 h bright light/10 h dark). After 4–21 days culture in the HYPONeX aqueous solution, the suspension was used for each experiment during the circadian time (CT) 10–14 h.

**Fig 1:**
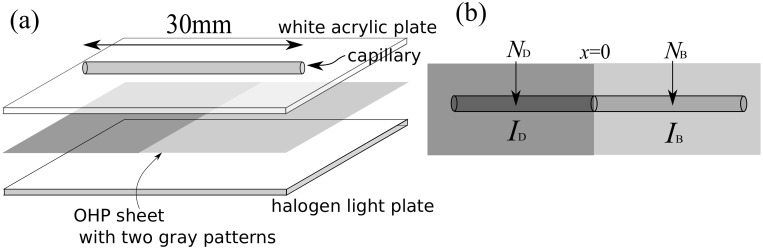
(a) Experimental setup. (b) Symbols for the numbers of cells and for the photo flux densities in the left and right part of the setup.

The flux of the cell density was measured under a light field with two different intensity regions (section 3). The HYPONeX culture was diluted to 2.50 × 10^5^ cells mL^−1^ in cell density and was put into a capillary glass (volume *V* = 1*μL*, length = 30 mm, inner radius = 0.10 mm). The capillary glass with the cell culture was illuminated for 60 min from below with a halogen flat light source (YAHATA, Megalight 100) through a white acrylic plate (1 mm in thickness) to diffuse the light (Fig 1(a)). Two regions with different light intensities were prepared using photo-mask of an OHP sheet on which a grayscale mask was printed using a laser printer (Canon MF8350). The photo flux density (PFD) at the observation point, *I*, was measured with the illumination photometer (Deltaohm, HD2302.0) on the white acrylic plate. The difference in PFD between the darker region (*I*_*D*_) and the brighter region (*I*_*B*_) was varied from 0.5 to 2.0 *μ*mol m^−2^ s^−1^ while keeping the average intensity (*I*_*D*_ + *I*_*B*_)/2 constant (Fig 1(b)). In the region near *x* = 0, the boundary of the light intensities, the light intensity exhibited the gradient. The maximum light gradient was -0.314 *μ*mol m^−2^ s^−1^ ⋅ *μ*m^−1^. To count the cell numbers contained in the darker (*N*_*D*_) and brighter (*N*_*B*_) parts, the capillary glass was cut at the center and the culture was removed onto a glass plate before rinsing the inside of the capillary glass once with distilled water. Then, the number of cells was counted in the photograph taken from the top of the glass plate with a micro lens (Raynox Universal Adapter UAC 3500) attached to the digital camera (GC-PX1, JVC, Japan). For each set of *I*_*B*_ − *I*_*D*_ and *I*_*B*_ + *I*_*D*_, ten experiments were performed. Although a normalized difference in two regions was used in later analysis, the distribution for *N*_*D*_ and *N*_*B*_ were characterized by 60.30 ± 19.41 and 151.80 ± 22.75 (mean ± standard deviation) for the case (*I*_*D*_ + *I*_*B*_)/2 = 1.25 *μ*mol m^−2^ s^−1^ and *I*_*B*_ − *I*_*D*_ = 1.5 *μ*mol m^−2^ s^−1^, for example.

**Fig 2:**
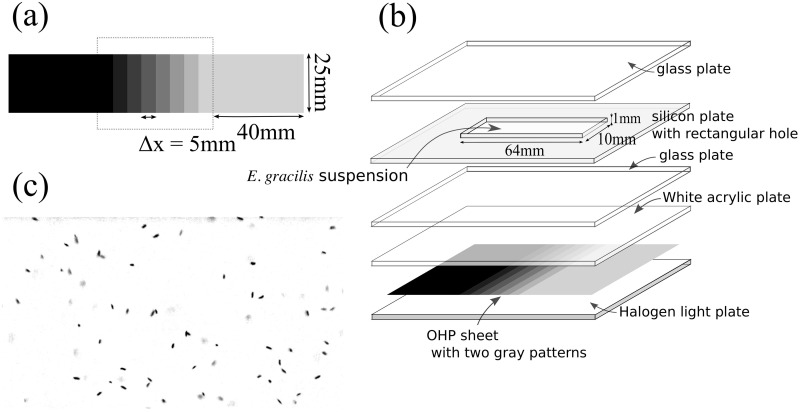
(a) Grayscale pattern on the OHP sheet. (b) Experimental setup. (c) An example of photograph (*I* = 23.25 *μ*mol m^−2^ s^−1^. 84 individuals are shown).

Steady-state cell density was estimated under illumination with a gradient of light intensity (section 4). Stepwise light intensities were generated by a photomask with an OHP sheet. Two kinds of discrete patterns were prepared as follows: The first pattern had eight steps and the light intensity increased from 1.47 *μ*mol m^−2^ s^−1^ to 25.76 *μ*mol m^−2^ s^−1^ with 3.47 *μ*mol m^−2^ s^−1^ in step with the rectangular regions (5 mm in width, 25 mm in height) (Sheet A; Fig 2(a), inside the dotted rectangle). The second pattern had seven steps and the light intensity increased from 1.47 *μ*mol m^−2^ s^−1^ to 8.41 *μ*mol m^−2^ s^−1^ with 1.16 *μ*mol m^−2^ s^−1^ in step (Sheet B). The patterns were sandwiched with two wider rectangular regions (40 mm in width, 25 mm in height) and the grayscale of each wide rectangular pattern was the same as that of the adjacent rectangular to eliminate boundary effects.

A silicon plate with 1 mm thickness with a rectangular hole was prepared and was sandwiched with glass plates to generate a Hele-Shaw cell (64 mm in width, 10 mm in height, and 1 mm in thickness). The HYPONeX culture was diluted to 0.50 × 10^5^ in cell density and was sealed into the Hele-Shaw cell. The Hele-Shaw cell was illuminated from below with the halogen flat light through the photo-mask and white acrylic plate (Fig 2(b)). After 2 h illumination, the cells in each rectangular region were photographed using the same optics as in the previous experiment. The optics was mounted on an apparatus that allows horizontal motion mechanically so that the height of focus was the same for all the regions during one scanning sequence. Also, the height of focus was carefully chosen to avoid near-boundary regions. The photographed region comprised a rectangle (2.9 mm × 2.2 mm) in the central part of each rectangular region and then the number of the cells was counted. We prepared two photographs taken with the interval of one second, converted them to grayscale images (Fig 2(c)), and counted the dark spots that moved between two images. For each density region, ten measurements were performed. We note that the width of the rectangular region was chosen so that it is larger than both the thickness of the Hele-Shaw cell and the width of the photograph region.

## 3 Cell density flux owing to the light gradient

In the first experiment (Fig 1), if *N*_*B*_ and *N*_*D*_ depend on the difference in the light intensity, *I*_*B*_ − *I*_*D*_, the cell density flux has another factor depending on the light environment. Here we modeled the population motion rather than individual motion. According to the result by Giometto et al. [5] that the cell density distribution satisfies a diffusion equation, we assumed that the cell density flux *J* was the sum of the diffusion effect and an extra term, *J*_*p*_, which was unknown at that moment, as follows.

$$J=J_{p}-D\frac{dn}{dx}.$$ (1)

Our purpose in this experiment was to clarify whether *J*_*p*_ depends on the light intensity difference, Δ*I* = *I*_*B*_ − *I*_*D*_, and to quantify the dependence on Δ*I*. For this purpose, the following formula was used. In the steady state, *J* = 0. We integrated Eq (1) over an interval [−*ε*, *ε*] (where 2*ε* is the characteristic width of the transition layer of the light intensity on the both sides) to obtain

$$0=\int_{-\varepsilon }^{\varepsilon }Jdx=\int_{-\varepsilon }^{\varepsilon }J_{p}dx-D\left(n\left(\varepsilon \right)-n\left(-\varepsilon \right)\right).$$ (2)

Regarding *n*(*ε*), *n*(−*ε*) as the mean cell density in the bright region and that in the dark region, *N*_*B*_/(*V*/2), *N*_*D*_/(*V*/2), respectively, we obtained

$$\int_{-\varepsilon }^{\varepsilon }J_{p}dx=\frac{2D(N_{B}-N_{D})}{V}.$$ (3)

Thus the averaged flux in the interval [−*ε*, *ε*], $\bar{J_{p}}$, is represented as

$$\bar{J_{p}}=\frac{1}{2\varepsilon }\int_{-\varepsilon }^{\varepsilon }J_{p}dx=\frac{D\Delta N}{\varepsilon V},\;\Delta N=N_{B}-N_{D}$$ (4)

**Fig 3:**
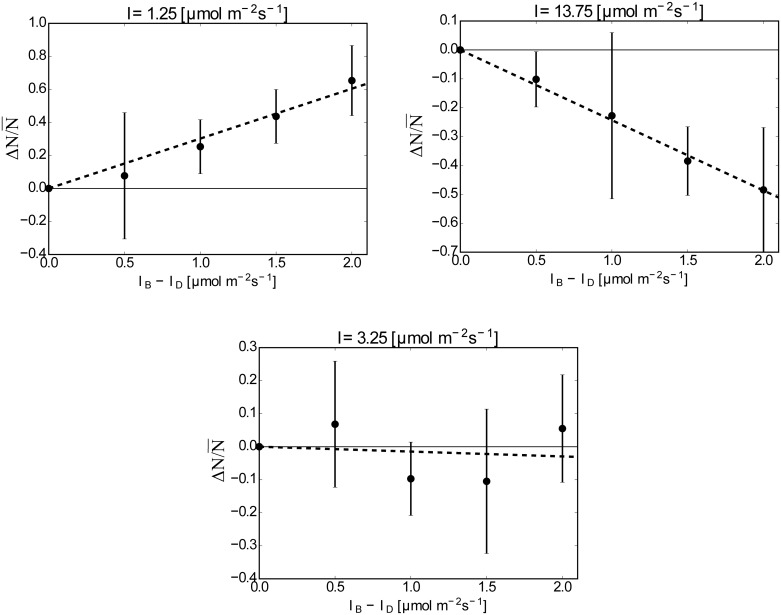
$\Delta N/\bar{N}$ vs. Δ*I* for *I*_mean_ = 1.25, 3.25, 13.75 *μ*mol m^−2^ s^−1^. **The averaged values and the standard deviations are shown by points and bars**. **Fitted lines are drawn by broken lines**. **The slopes are**: 0.302 ± 0.0252 **for** *I*_mean_ = 1.25 *μ*mol m^−2^ s^−1^, −0.0147 ± 0.0226 **for** *I*_mean_ = 3.25 *μ*mol m^−2^ s^−1^ **and** −0.243 ± 0.0217 **for** *I*_mean_ = 13.75 *μ*mol m^−2^ s^−1^.

This formula gives the relationship between $\bar{J_{p}}$ and Δ*N*, and Δ*N* was measured experimentally. We measured Δ*N* for different Δ*I* while keeping the mean light intensity *I*_mean_ = (*I*_D_ + *I*_B_)/2. The results are summarized in Fig 3, where $\Delta N/\bar{N}\;\left(\bar{N}=N_{D}+N_{B}\right)$ is plotted for Δ*I* for *I*_mean_ = 1.25, 3.25 and 13.75 *μ*mol m^−2^ s^−1^. The cell number in the capillary, (*N*_*D*_ + *N*_*B*_), ranged from 135 to 319, whereas the number was estimated as 250 cells from the mean cell density of the culture. When *I*_mean_ = 1.25 *μ*mol m^−2^ s^−1^, the mean values of *N*_*B*_ for each value of Δ*I* were always larger than those of *N*_*D*_; that is, the individuals tended to accumulate in the brighter region on average. On the other hand, when *I*_mean_ = 13.75 *μ*mol m^−2^ s^−1^, the mean values of *N*_*B*_ were always smaller than those of *N*_*D*_ and the individuals tended to accumulate in the darker region on average. These two cases show that the horizontal light gradient causes a bias of the cell density and that the direction of the cell density flux depends on *I*_mean_. An almost neutral case, *I*_mean_ = 3.25 *μ*mol m^−2^ s^−1^, is also shown, where the average values of *N*_*D*_ and *N*_*B*_ were almost the same even though the light gradient was present.

Furthermore, Fig 3 suggests that $\Delta N/\bar{N}$ is proportional to Δ*I*:

$$\frac{\Delta N}{\bar{N}}=k\Delta I⟺\Delta n=2k\bar{n}\Delta I,$$ (5)

where *k* is a constant determined by the mean light intensity and $\Delta n=n\left(\varepsilon \right)-n\left(-\varepsilon \right)=\left(N_{B}-N_{D}\right)/\left(V/2\right),\bar{n}=\frac{1}{2}\left(n\left(\varepsilon \right)+n\left(-\varepsilon \right)\right)=\left(N_{B}+N_{D}\right)/V$. Then, we could determine the flux $\bar{J_{p}}$ as

$$\bar{J_{p}}=\frac{kD\bar{n}\Delta I}{\varepsilon }=2kD\bar{n}\bar{\nabla I},$$ (6)

where $\bar{\nabla I}=\frac{1}{2\varepsilon }\int_{-\varepsilon }^{\varepsilon }\frac{dI}{dx}dx=\frac{\Delta I}{2\varepsilon }$ is the averaged light intensity gradient.

Taking the limit *ε* → 0, we obtained the following form of *J*_*p*_:

$$J_{p}=Df\left(I\right)n\frac{dI}{dx},$$ (7)

where the coefficient 2*k* was replaced with a function of the light intensity, *f*(*I*).

## 4 Function of the photomovement owing to the light gradient

Next, we estimated *f*(*I*) experimentally. We used the following formula. By Eq (1) and the form of *J*_*p*_ (Eq (7)), we assumed the following form of the cell density flux in one-dimensional case as

$$J=D\left(f\left(I\right)n\frac{\partial I}{\partial x}-\frac{\partial n}{\partial x}\right).$$ (8)

In the steady state, *J* = 0. Then,

$$\frac{1}{n}\frac{\partial n}{\partial x}=f\left(I\right)\frac{\partial I}{\partial x}.$$ (9)

Thus, we could estimate *f*(*I*) by measuring the cell density distribution in the steady state, *n*(*x*), under the prescribed light intensity distribution *I*(*x*). For this purpose, the experiment in Fig 2 was performed.

The total field of the light intensity can be regarded as stepwise, whereas the average linear light gradient *dI*/*dx* = *γ* in the sheet A was *γ* = *γ*_*A*_ = 0.694 *μ*mol m^−2^ s^−1^ ⋅ mm^−1^ and that in the sheet B was *γ* = *γ*_*B*_ = 0.231 *μ*mol m^−2^ s^−1^ ⋅ mm^−1^. In the following, we assumed that *dI*/*dx* = *γ*.

Because the cell density was dilute and the suspension depth was shallow, we could exclude the effect of self-shading of *E. gracilis*; in particular, no bioconvection occurred. In addition, the depth of focus of the camera, *a*, was small compared with the depth of the Hele-Show cell, *d*. Thus, the counted number in each region, *N*, was estimated by the cell density multiplied with *a* and the observation region *A*: *N* = *nAa*. In the actual measurements, we used the averaged frequency distribution of *N*, *n*′, which is also proportional to *n*. From these measured values, we could obtain *f*(*I*) as

$$f\left(I\right)=\frac{1}{\gamma n}\frac{\partial n}{\partial x}=\frac{1}{n^{\prime }}\frac{\partial n^{\prime }}{\partial I}.$$ (10)

**Fig 4:**
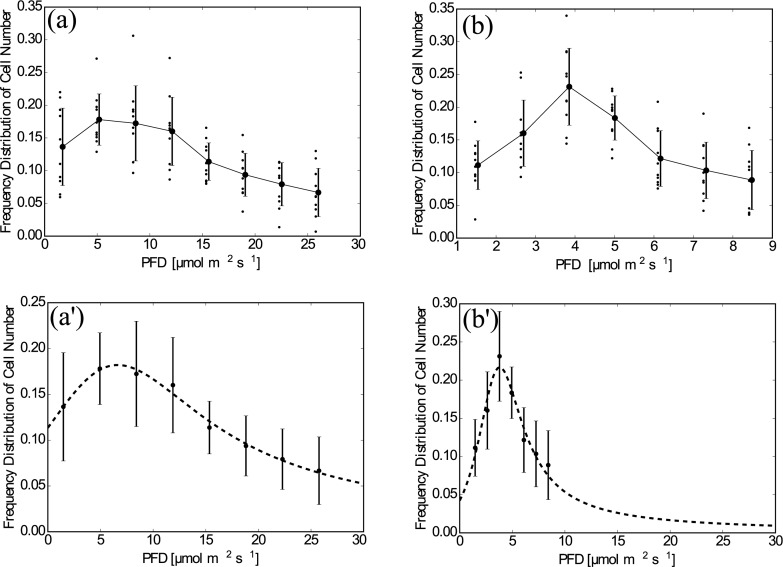
Plotted data of *n*′(*x*). (a) Sheet A, raw data. The averaged values and the standard deviations are shown by points and bars, which are slightly shifted to the right to avoid overlapping with the raw data. (b) As in (a) but for Sheet B. (a)’ Sheet A, The curve fitted by Eq (11). The averaged values and the standard deviations are shown by points and bars. (b)’ As in (a)’ but for Sheet B and the horizontal range was set the same as for (a)’.

Fig 4 shows the frequency distribution of the counted numbers of cells, *n*′. Through all the experiments, no global flow or self-shading of individuals was observed. The results for sheet A show a slow decay (*I*^−1^) of *n*′ when *I* > 15 *μ*mol m^−2^ s^−1^. There are single peaks in both graphs; in particular, *I* = 3.80 *μ*mol m^−2^ s^−1^ for sheet B. These results show that the *E. gracilis* prefers a certain intensity of light and moves according to the light gradient. The distribution using sheet B suggests that *n*(*x*) has a large variation in the region *I* < 10 *μ*mol m^−2^ s^−1^; the data for Sheet A is smoothed out in this region.

The obtained distributions qualitatively agree with the result obtained when considering cell density flux, where *E. gracilis* moved to the brighter region when *I* = 1.25 *μ*mol m^−2^ s^−1^ whereas it preferred the darker region when *I* = 13.75 *μ*mol m^−2^ s^−1^.We assumed that the measured value of the frequency distribution of the cell number, $n_{f}^{\prime }$, which is proportional to the cell density distribution, was represented by a fractional function of the following form:

$$n_{f}^{\prime }\left(I;a,b,c,d\right)=\frac{a+bI}{c+dI+I^{2}},$$ (11)

where the independent variable was replaced with *I* using the relationship *I*(*x*) = *γx*. This form was selected so that $n_{f}^{\prime }\left(I;a,b,c,d\right)∼I^{-1}$ when *I* was large, as reflected from the result of Sheet A (Fig 4(a)).

Fitted values of *a*, *b*, *c* and *d* obtained by the Levenberg-Marquardt method as well as the light intensity giving the peak of the fitted curve, I*, are listed in Table 1 for both cases of Sheet A and Sheet B. The fitted function takes the maximum at *I* = *I*_*c*_ = 3.8 *μ*mol m^−2^ s^−1^ for Sheet B.

**Table 1:** Tables of fitted parameters. ‘Merged model’ stands for the merged model for Sheet A and Sheet B.

	Sheet A	Sheet B	merged model
*a*	1.327448 × 10^1^	7.596000 × 10^−1^	1.980453 × 10^0^
*b*	8.988007 × 10^−1^	1.974052 × 10^−1^	1.018627 × 10^0^
*c*	1.169945 × 10^2^	1.797669 × 10^1^	1.565095 × 10^1^
*d*	−8.334796 × 10^0^	−6.699378 × 10^0^	−6.001808 × 10^0^
*I*_*c*_	6.636932	3.804651	3.632495
α*	-	-	3.96

These two experiments give the cell density distribution in different light regions; the decay law in the bright region was obtained by the experiment using Sheet A and the detailed distribution near the maximum of *n*′ was examined by the experiment using Sheet B. Here we remark that the cell density *n*(*I*) was expected to be proportional to $n_{f}^{\prime }\left(I;a,b,c,d\right)$; however, their proportional coefficients are not the same at a particular value of *I* (or *x*) because of the following two reasons: (1) $n_{f}^{\prime }\left(I;a,b,c,d\right)$ (and the depicted data) do not represent the cell density itself but a normalized value by using the total count of the number of cells. (2) The cell number distribution depends on both the mean cell density and the light distribution. Therefore, we cannot merge the results for Sheet A and Sheet B directly.

To obtain the function *f*(*I*) using these data, we did not need the absolute value of the cell density because the proportionality does not change *f*(*I*): *f*(*I*) is invariant under the transformation *n*′ → *αn*′(*α* is a constant). By utilizing this fact, we estimated the number distribution function covering the whole experimental region as follows. Let us define the data set of the light intensity and the cell density in the experiments using Sheet A and Sheet B as *S*_*A*_ and *S*_*B*_, respectively. Here, *S*_*A*_ and *S*_*B*_ are $\left\{\left(I_{i},n_{i}^{\prime }\right)|i=1,2,\cdots ,M\right\}$, where *M* is the number of data. We define a critical light intensity separating the decaying region and the detailed region by *I* = *I*_*b*_ = 10*μ*mol m^−2^ s^−1^. We merge *S*_*A*_ and *S*_*B*_ with consideration that each set has an uncertain factor of the cell density, which results from the different light gradient region. Without loss of generality, we can assign the uncertain factor to the data set *S*_*A*_ alone. Now, the merged data set *S*(*α*) is defined as

$$S\left(\alpha \right)=S^{\prime }{\left(\alpha \right)}_{A}\cup S_{B}^{\prime },$$ (12)

$$S^{\prime }{\left(\alpha \right)}_{A}=\left\{\left(I,\alpha n^{\prime }\right)|\left(I,n^{\prime }\right)\in S_{A},I<I_{b}\right\},\;S_{B}^{\prime }=\left\{\left(I,n^{\prime }\right)|\left(I,n^{\prime }\right)\in S_{B},I>I_{b}\right\}.$$ (13)

**Fig 5:**
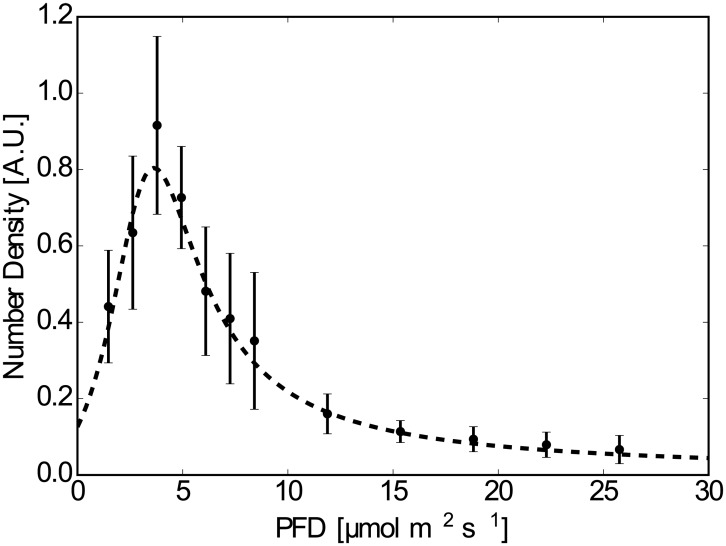
Fitted function to the data set *S*(α*).

Fitting the function $n_{f}^{\prime }$ to the data set *S*(*k*) is performed as follows. We define the error of fitting as a function of *α* as:

$$E\left(\alpha ;a,b,c,d\right)=\sum_{\left(I,n^{\prime }\right)\in S\left(\alpha \right)}{\left(\log\left({\tilde{n}}_{f}^{\prime }\left(I;a,b,c,d\right)\right)-\log\left(n^{\prime }\right)\right)}^{2},$$ (14)

$${\tilde{n}}_{f}\left(I;a,b,c,d\right)=\frac{a+bI}{c+dI+I^{2}},$$ (15)

where ${\tilde{n}}_{f}$ is a function proportional to the cell density distribution. Here we adopted the difference between the logarithm of the data and the functions to weight the data in the decaying region, so that the tail part of the distribution would also contribute to the error estimation. To find the set of *α* and (*a*, *b*, *c*, *d*) to minimize *E*(*α*; *a*, *b*, *c*, *d*), we found the parameters (*a*, *b*, *c*, *d*) to minimize *E*(*α*; *a*, *b*, *c*, *d*) for a given value of *α*. The value of the minimized error was defined as $\tilde{E}\left(\alpha \right)$. Then, we find the minimizer *α* = α* such that $d\tilde{E}/d\alpha =0$ to determine the best parameters of *α* and (*a*, *b*, *c*, *d*). In practical application, we calculate $\tilde{E}\left(\alpha \right)$ in the interval 1 ≤ *α* ≤ 5 with increments of 0.01 and find α* so that $\tilde{E}\left(\alpha^{*}\right)$ is minimum. The results are listed in Table 1(Right), where α* = 3.96 and I* = 3.63 were obtained. The fitted function for the dataset *S*(*k*) is shown in Fig 5.

**Fig 6:**
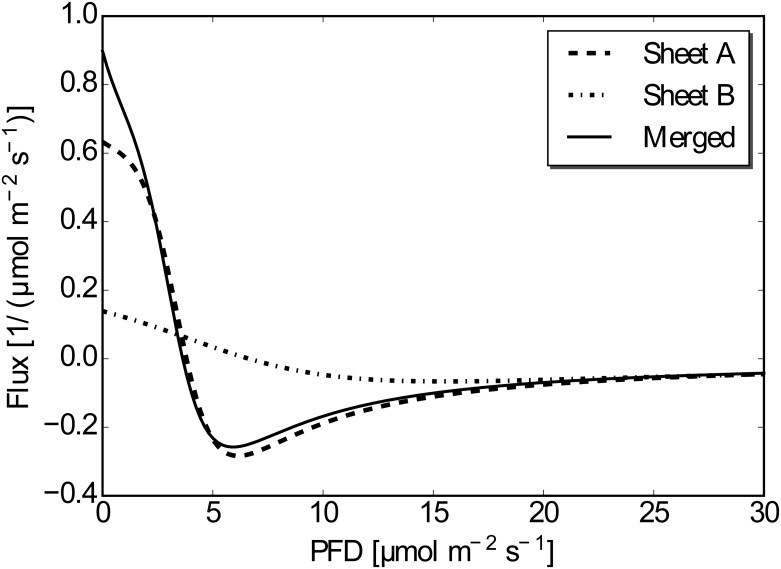
The function *f*(*I*) for the data set *S*_*A*_, *S*_*B*_, *S*(α*).

Substituting functions ${\tilde{n}}_{f}^{\prime }$ and $n_{f}^{\prime }$ to *n*′ in Eq (10), we calculate the function *f*(*I*). Here we remark again that the constant uncertainty does not affect the calculation of *f*(*I*), so both ${\tilde{n}}_{f}^{\prime }$ and $n_{f}^{\prime }$ can be used to calculate *f*(*I*). The calculated functions of *f*(*I*) are shown in Fig 6. The overall shape of the function for Sheet A is similar to that for the merged one except for smaller values of the light intensity. The shape of the function for Sheet B is similar to the merged one for larger values of the light intensity, whereas it deviates from the shape for the merged one owing to coarse data. The value of *f*(*I*) should be identical to the value of 2*k* obtained when considering cell density flux; *k* = 0.302, −0.0147, −0.243 for *I*_mean_ = 1.25, 3.25 and 13.75*μ*mol m^−2^ s^−1^, respectively. The value *k* = 0.302 (*I*_mean_ = 1.25 *μ*mol m^−2^ s^−1^) deviates from the result, because it is outside the region of the measured value and the fitted function thus cannot well represent the data. The other two values show reasonable agreement with the estimated value of *f*(*I*).

The sign of *f*(*I*) changes at *I* = *I*_*c*_ by Eq (10). Thus, if the light intensity is less than *I*_*c*_, *E. gracilis* tend to move toward a brighter region. If the light intensity is larger than *I*_*c*_, *E. gracilis* tend to move toward a darker region. However, the function *f*(*I*) takes a value of almost zero where I is large (e.g., 15 *μ*mol m^−2^ s^−1^), so *E. gracilis* is almost neutral with respect to the light gradient if the mean light intensity is large enough.

## 5 Concluding Remarks

In this study, we performed two experiments to examine the distribution of cell density of a suspension of *E. gracilis* under a light intensity gradient. In the first experiment, the flux of the cell density was measured and the result suggested that the flux was proportional to the light intensity gradient and the cell density. The proportional coefficients could be both positive and negative, depending on the mean light intensity. In the second experiment, the coefficient was estimated as a function of the light intensity. The obtained results showed that *E. gracilis* tended to accumulate toward the region with a particular light intensity (approximately 3.8 *μ*mol m^−2^ s^−1^) when the suspension was illuminated from below in such a manner that a linear gradient of light intensity was generated, although the critical value for individuals might have a diversity. In these experiments, the major phototaxis effect was vertical because of vertical illumination, which is a different setup from that used by Giometto et al., in which point light source was used and the mean light vector was tilted [5].

According to the experiment of localized bioconvection using *E. gracilis* [15, 16], we took the typical cell density as *ρ* = 10^6^ cells/mL and the depth of the suspension as *d* = 5mm. The absorption coefficient of the suspension of *E. gracilis*, *k*_*a*_, is 2.84 × 10^−10^ m^2^/cells (S. Noda, Master thesis in Hiroshima University, 2012). Using the Lambert-Beer law, we can estimate the light intensity at the top of the suspension illuminated below with the light intensity *I*_0_, *I*, as

$$I=I_{0}\exp\left(-k_{a}\rho d\right)=0.24I_{0}$$

If we take *I*_0_ = 30 *μ*mol m^−2^ s^−1^, corresponding to about 1200 lx, we obtain *I* = 7.2 *μ*mol m^−2^ s^−1^, which is above the threshold *I*_*c*_. Therefore, if there is perturbation in the corresponding uniform cell density distribution in the whole region, the perturbation grows owing to the light gradient. This instability does not depend on the wavelength of the perturbation and is independent of the Rayleigh-Taylor instability that causes many kinds of (non-localized) bioconvection. In this sense, the photomovement owing to the light gradient might represent the major reason of the localization.

The mechanism of the accumulation is still an open question. Because *E. gracilis* has a single paraflagellar body, which acts as a sensory organ, an individual of *E. gracilis* cannot detect an instantaneous light gradient. However, the present results suggest that *E. gracilis* behave as if they can sense the light gradient. A theoretical model of the biochemical kinetic processes of photoreceptors has been proposed to reveal the mechanism of photophobic reactions [7]. However, the present characteristics of *f*(*I*) cannot be completely explained through this model alone. Several alternate candidates for the mechanism are listed below.

The first candidate is a phototaxis-based mechanism. In effect, *E. gracilis* might integrate the information of the scattering light in a light gradient environment. In the current setup, we used an acrylic plate to diffuse the light so that an individual cell in the light gradient environment would detect scattering light vectors not only from directly below but also from obliquely below. Light vectors from obliquely below are tilted and their intensities are not uniform. Thus, an integration of the phototactic response of different magnitudes might lead to the observed response to the light gradient. This scenario would be confirmed if the phototaxis response changes at the same value of light intensity as that at which the function *f*(*I*), the response function to the light gradient, takes zero, i.e. 3.8 *μ*mol m^−2^ s^−1^, under the same light condition. The critical value of the change of the phototaxis behavior determined by Häder et al. was around 250 lx (= 1.05 Wm^−2^) [4]. The exact conversion of the irradiance to the photo flux density requires the spectrum of the source light, which is not available. An estimation under the assumption that the light had a single peak at 470 nm gives that 1.05 Wm^−2^ = 4.1 *μ*mol m^−2^ s^−1^. We stress that this value gives only an order of the photo flux density; however, it is notable that it has the same order as our critical value.

The second possibility is a photophobic-based mechanism. When an individual cell of *E. gracilis* swims under the light gradient environment, the light intensity changes according to the swimming path, which might cause a change of swimming direction. According to the experiment by Matsunaga et al. [8], an increase or decrease of light intensity by a few *μ*mol m^−2^ s^−1^ change might increase the occasion of velocity change in a statistical sense. The value of light intensity is also the same order as the critical value of our experiments, although their experiments examined the behavioral change between light and the no light conditions.

To understand how the kinematical mechanism might achieve a gradient-sensing, the chemotaxis mechanism in *Escherichia coli* and some other bacteria is suggestive. *E. coli* and some other bacteria show similar phenomena to the step-down (step-up) photophobic reaction of *E. gracilis* [22–27]. Here, *E. coli* exhibits a temporal increase of its tumbling rate for changing its swimming direction, termed adaptation, consequent to sudden decreases (increases) in the concentrations of a chemoattractant (chemorepellent). The chemotaxis of *E. coli* has also been extensively investigated experimentally and theoretically [22–32]. Recently, a theoretical study using a model of chemoreceptor biochemical kinetics derived the experimentally observed form of a function, which depends on the background chemical concentration relationship to the chemotaxis sensitivity; this function corresponds to *f*(*I*) in the present study. [32].

Our experiments suggest that the flux of a suspension of *E. gracilis* depends on the light gradient. The measurements give other aspects of collective motion of *E. gracilis*, which might be useful to understand the collective behavior of a suspension of *E. gracilis*, especially the spatially localized bioconvection patterns.
